# Systematic analysis of structural disorder in the minimal proteome of *Mycoplasma pneumoniae*

**DOI:** 10.1128/spectrum.00968-25

**Published:** 2025-06-18

**Authors:** Uberto Pozzoli, Diego Forni, Federica Arrigoni, Alessandra Mozzi, Rachele Cagliani, Luca De Gioia, Manuela Sironi

**Affiliations:** 1 Scientific Institute IRCCS E. MEDEA, Computational Biology Unit, Bosisio Parini, Italy; 2 Department of Biotechnology and Biosciences, University of Milan-Bicocca 9305, Milan, Italy; 3 School of Medicine and Surgery, University of Milano-Bicocca, Monza, Italy; Griffith University-Gold Coast Campus, Gold Coast, Australia

**Keywords:** *Mycoplasma pneumoniae*, intrinsically disordered region, AlphaFold, structural disorder, ensemble features

## Abstract

*Mycoplasma pneumoniae* (Mpn, class *Mollicutes*) is both an important human pathogen and a model organism. We performed a proteome-wide investigation of intrinsically disordered regions (IDRs) in Mpn. Compared to other bacteria, a considerable fraction of the Mpn proteome (17%) is embedded in IDRs, which are abundant in membrane, non-essential proteins, as well as in proteins that mediate cytoadherence and virulence. Notably, proteins that form the attachment organelle, a specialized structure, are particularly rich in IDRs. Likewise, analysis of protein architectures indicated that some Mollicute-specific domains are preferentially associated with IDRs. Perusal of proteome-wide data also revealed that, as in eukaryotes, structural disorder associates with higher protein degradation rates and that Mpn IDRs are preferential targets of phosphorylation. When we investigated the ensemble features for Mpn IDRs, we used two predictors and benchmarked the results using coarse-grained simulations. We found that ensemble properties are mediated by similar sequence features as in eukaryotes, so that compact IDRs tend to have high residue stickiness, high hydropathy decoration, and few charged residues. We also found that IDRs in attachment organelle proteins are particularly extended and display high conformational entropy. We suggest that these features are exploited for motility through the generation of an entropic force. In summary, our results suggest that structural disorder contributes to very specialized functions in Mpn. Our data also highlight the functional relevance of IDRs, as the minimal proteome of this model organism displays a considerable level of structural disorder.

**IMPORTANCE:**

We performed a proteome-wide investigation of intrinsically disordered regions (IDRs) in *Mycoplasma pneumoniae* (Mpn, class *Mollicutes*). A considerable fraction of the Mpn proteome (17%) is embedded in IDRs, which tend to be associated with Mollicute-specific domains and are abundant in membrane, non-essential proteins, as well as in proteins that mediate cytoadherence and virulence. As in eukaryotes, structural disorder associates with higher protein degradation rates, and Mpn IDRs are preferential targets of phosphorylation. The ensemble properties of Mpn IDRs are mediated by similar sequence features as in eukaryotes, and IDRs in attachment organelle proteins display high conformational entropy. We suggest that this feature is exploited for motility through the generation of an entropic force. In summary, we show that structural disorder contributes to very specialized functions in Mpn. Our data highlight the functional relevance of IDRs, as the minimal proteome of this model organism displays a considerable level of structural disorder.

## INTRODUCTION

Intrinsically disordered proteins (IDPs) and protein regions (IDRs) do not adopt a stable three-dimensional structure (3D), but rather exist in conformational ensembles—that is, collections of energetically accessible, rapidly interconverting structures (1). Molecular interactions within an IDR determine the overall properties of the conformational ensemble, such as its compaction or extension. In turn, conformational features are often linked to functional properties (2, 3). Thus, IDRs are generally described by “sequence-ensemble-function” relationships. Structural disorder is common in the proteomes of living organisms and viruses. In eukaryotes, IDRs cover up to 40% of the proteome and have been implicated in a variety of cellular processes (1). In viruses, large variations in the IDR fraction are observed among, but also within, viral families (4–7). In viral proteomes, IDR-containing proteins often contribute to host-virus interactions (4, 5, 8–10). In bacteria and archaea, IDRs are less abundant, but they were reported to be enriched in proteins that contribute specific functions such as sporulation, septum formation, cell wall biosynthesis, and flagellum-dependent motility (1, 5, 11–13). Analysis of individual IDRs in bacterial proteins also revealed that they may be important for mechanisms related to adaptation and pathogenesis such as toxin-antitoxin systems, protection against antimicrobial peptides, and biofilm formation (14–18). Despite these insights, systematic analyses of IDRs in bacterial systems are missing, and the functions of these regions remain largely unexplored. We thus aimed to fill this knowledge gap by focusing on the proteome of *Mycoplasma pneumoniae* (Mpn, order Mycoplasmoidales*,* class *Mollicutes)*, a relevant human pathogen and a model organism (19)*.* Among self‐replicating prokaryotes, Mpn has one of the smallest known genomes, which encodes fewer than 700 proteins (19, 20). The small genome of Mpn is a consequence of its adaptation to an obligate parasitic lifestyle, and the bacterium lacks a cell wall (20). Infection with Mpn can cause chronic human respiratory tract disease and pneumonia (21). Across age groups, Mpn is estimated to be responsible for up to 40% of cases of community-acquired pneumonia (21). In this study, we integrated the predictive power afforded by AlphaFold2, coarse-grained simulations, and deep learning tools to analyze conformational ensembles, as well as large-scale *in vitro* data to provide an overview of the functional features of IDRs in the Mpn proteome.

## RESULTS

### IDR prediction in Mpn proteins

We aimed to predict the overall IDR content of the Mpn proteome. To benchmark the prediction method, we retrieved information on all (*n* = 18) Mpn proteins with an experimentally solved structure from the PDB database (22) (Table S1). From these structures, we annotated gap regions—that is, regions that were not solved in the crystal and are thus likely to be disordered. Gaps longer than 29 amino acids were considered IDRs (3). We identified five IDRs in three proteins (15 proteins were fully folded). We next used two approaches for IDR prediction. Specifically, we applied Metapredict V2, a deep-learning-based method that combines different predictors to generate consensus disorder scores (23, 24), and we used an IDR prediction approach based on per-residue predicted local difference test (pLDDT) scores from AlphaFold2 models (3) (see Materials and Methods). In both cases, disordered regions shorter than 30 residues were not considered IDRs (3). We next calculated the sensitivity, specificity, and accuracy for the two methods based on the fractions of residues that were correctly or incorrectly (according to the structural data) classified as IDR/folded. Results indicated that the AlphaFold2-based method (sensitivity = 0.996, specificity = 0.941, accuracy = 0.943) performed better than Metapredict V2 (sensitivity = 0.577, specificity = 0.871, accuracy = 0.859).

### IDRs are enriched in membrane proteins that mediate Mpn cytoadherence

**Fig 1:**
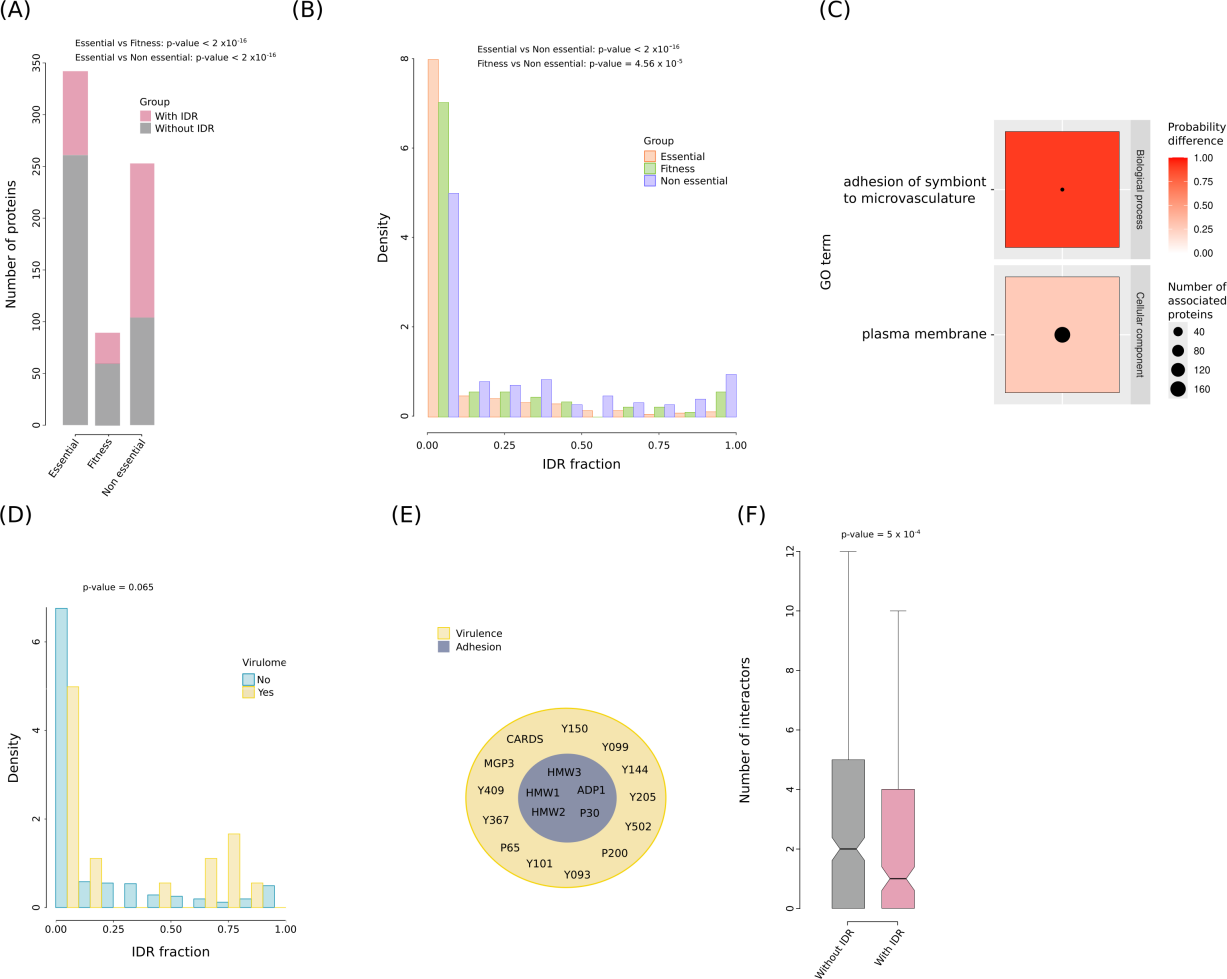
Function and interactions of IDRs in the Mpn proteome. (**A**) Comparison of the fraction of proteins with IDR in different essentiality categories. Fitness refers to proteins that have some impact on growth, but do not affect viability. *P*-values derive from Fisher’s exact tests. (**B**) Density histogram of IDR fraction (per protein) in the same classes. *P*-values are from Brunner-Munzel tests. (**C**) GO analysis of IDR fraction. Only significant terms are shown (*P*-value < 0.01 after FDR correction). The red scale represents the probability difference from Brunner-Munzel tests. The number of proteins associated with any term is represented by circle diameters. (**D**) Density histogram of IDR fraction in proteins that influence virulence and in those that do not (*P*-value from Brunner-Munzel test). (**E**) Venn diagram of proteins involved in virulence and of those associated with the GO term “adhesion of symbiont to microvasculature.” (**F**) Number of protein-protein interactions (PPIs) for Mpn proteins with or without IDRs (*P*-value from Brunner-Munzel test).

Given the results above, we used the AlphaFold2-based approach to investigate the IDR content in the reference proteome of Mpn strain M129 (686 proteins). We estimated that ~17.4% of residues in the Mpn proteome are embedded within IDRs and that 260 proteins (37.9%) have at least one IDR. These proportions are higher than those reported for most bacteria (25–27). We first asked whether IDRs are more common in bacterial essential proteins or in proteins that are dispensable for optimal growth. We thus retrieved data from a transposon insertion mutagenesis study that categorized Mpn proteins as essential, non-essential, or fitness (which have an impact on growth, but do not affect cell viability) (28). We found that 23.7% of essential proteins have at least one IDR, whereas this percentage amounts to 58.9% and 33.3% in non-essential proteins and proteins that affect fitness (Table S2). These differences are statistically significant (Fisher’s Exact Test, *P*-value <2 × 10^−16^) (Fig. 1A). In fact, when we compared the fraction of protein sequence in IDRs, this was significantly higher in non-essential proteins compared to the essential ones (Brunner-Munzel test, *P*-value <2 × 10^−16^) and to the ones that have an impact on growth (Brunner-Munzel test, *P*-value = 4.56 × 10^−5^) (Fig. 1B).

We next investigated whether IDRs are particularly abundant depending on Mpn protein function or cellular localization. We evaluated the significance of whether proteins associated with a given gene ontology (GO) term have a higher IDR fraction than proteins not associated with that term. Results indicated that proteins involved in adhesion to microvasculature and those located at the plasma membrane have a significantly higher IDR fraction than proteins that are not associated with these terms (Fig. 1C) (Table S2). Inspection of proteins associated with the GO term “adhesion of symbiont to microvasculature” indicated that all of them are components of the Mpn attachment organelle, a specialized structure that mediates adherence and gliding motility (29).

We next sought to explore IDR content in relation to virulence. We thus retrieved a list of 18 Mpn virulence factors using VirulenceFinder through the MicroScope website (30) (Table S2). Results indicated that virulence factors have a higher IDR fraction than proteins that do not contribute to virulence, although the statistical significance threshold was not reached, possibly because of the small sample size (Brunner-Munzel test, *P*-value = 0.065) (Fig. 1D). Because adherence is linked to Mpn pathogenic features, we found a considerable overlap among proteins identified in the GO analysis under the term “adhesion of symbiont to microvasculature” and those involved in virulence (Fig. 1E).

Finally, because IDRs are common in eukaryotic proteins that function as signaling hubs, we asked whether this was also the case in Mpn. We thus retrieved information about Mpn protein complexes from a previous work (31), and we counted the number of interactions that each protein establishes with other proteins (Table S2). We observed that proteins with IDRs have significantly fewer protein-protein interactions (PPIs) than proteins without IDRs (Brunner-Munzel test, *P*-value = 5 × 10^−4^) (Fig. 1F). Overall, these results indicate that, in Mpn, IDRs may mediate attachment to host cells and contribute to virulence. Conversely, they have a limited role in controlling housekeeping cellular functions and are unlikely to participate in protein complexes.

### Domains specific to Mollicutes are enriched in IDR-containing proteins

**Fig 2:**
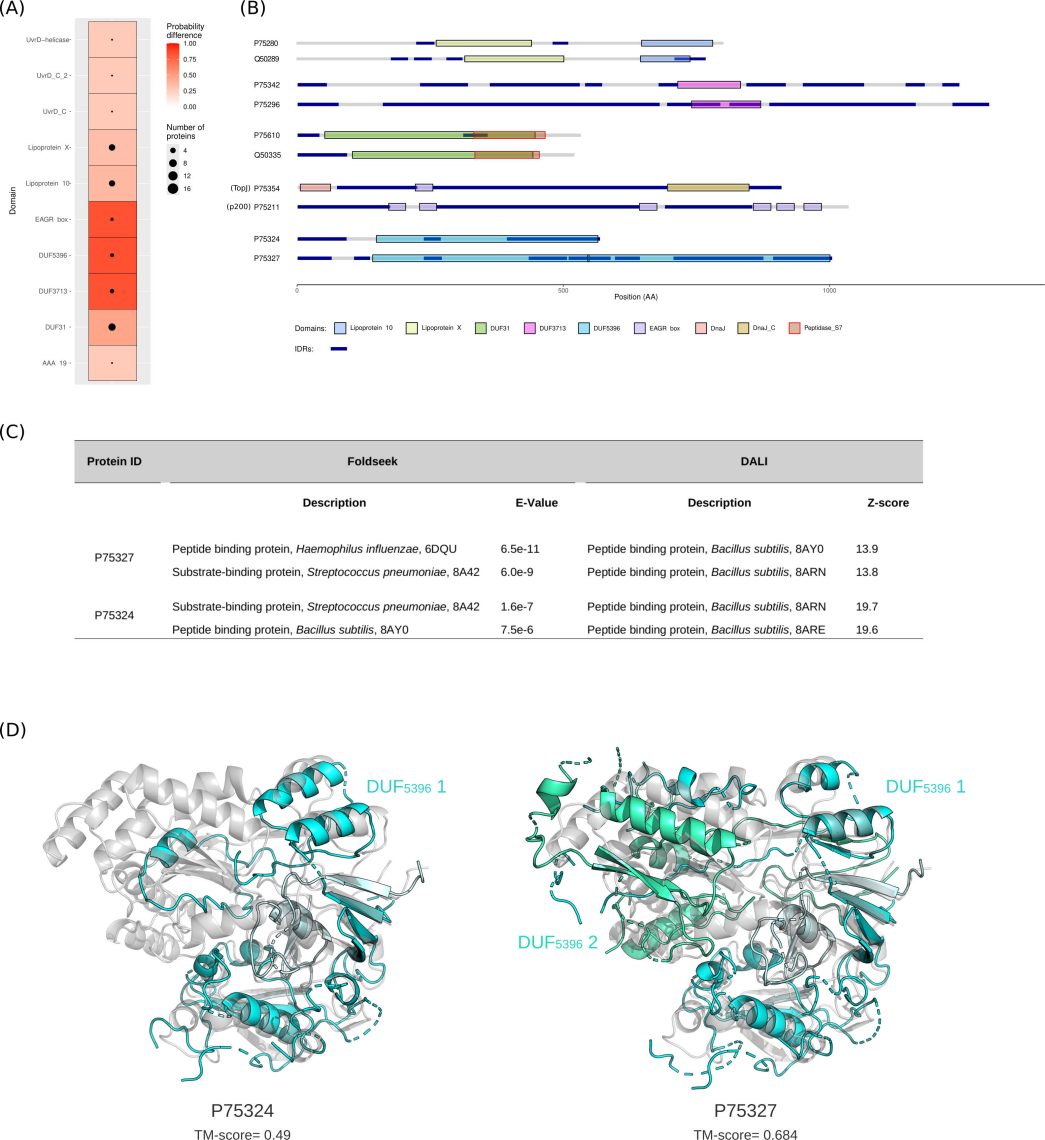
Analysis of protein domains associated with IDR fraction. (**A**) Protein domains associated with a high IDR fraction. Only significant terms are shown (*P*-value < 0.01 after FDR correction). The red scale represents the probability difference from Brunner-Munzel tests. (**B**) Domain structures of representative proteins carrying domains identified in the enrichment analysis. (**C**) Results of the structural homology searches. The AlphaFold-predicted structures of P75324 (AF-P75324-F1-v4) and P75327 (AF-P75327-F1-v4) were used to search the protein structure database using Foldseek and DALI. (**D**) Structural alignment (TM-align) of AF-P75324-F1-v4 and AF-P75327-F1-v4 with the solved structure of the OppA protein from *Bacillus subtilis* (PDB ID: 8arn). TM scores are also reported.

We next wished to investigate the architecture of IDR-containing proteins in terms of domain representation. We thus used SMART (Simple Modular Architecture Research Tool) to identify and annotate protein domains in the Mpn proteome. We identified a total of 563 different domains, but only 125 of these occurred in more than one protein. We thus limited the analysis to domains that occurred at least twice, and we evaluated the significance of whether proteins with a given domain have a higher IDR fraction than proteins without the same domain. We found statistically significant associations with IDR fraction for 10 domains (Fig. 2A). Of these, four (AAA_19, UvrD−helicase, UvrD_C_2, and UvrD_C) appear in very few proteins that have a role in DNA binding and repair. Two additional domains (lipoprotein X and 10) are characteristics of prokaryotic lipoproteins (32) and often occur in tandem in the same protein (Fig. 2B) (Table S3). In Mpn, these proteins were classified as family 2 lipoproteins (33). According to InterPro annotations, the remaining four domains are only found in Mollicutes. One of them, the EAGR box domain, has been implicated in gliding motility (34). In fact, it appears in three proteins of the attachment organelle (topJ, p200, and high molecular weight protein 1 [HMW1]) (Fig. 2B). The three other domains are classified as DUF (domains of unknown function). However, DUF31 is a putative serine protease, and in fact, it often overlaps with a peptidase S7 domain (Fig. 2B). Interestingly, the DUF31 domain was detected in an IgG protease encoded by *Mycoplasma mycoides* (but not Mpn), and analysis of Mollicute genomes showed that it is more common in species that are pathogenic to humans or other animals compared to plant pathogens (35). DUF3713 and DUF5396 have no known function. Whereas the former is relatively short, DUF5396 domains cover a substantial portion of two Mpn proteins (P75324 and P75327, both annotated in UniProt as uncharacterized lipoproteins), also overlapping with IDRs (Fig. 2B). To gain insight into the possible function of DUF5396, we used methods that search protein structure databases for structural homologs (36, 37). The top-scoring hits of both Foldseek and DALI were bacterial proteins that function as oligopeptide transporters (Fig. 2C). Pairwise structural alignments using TM-align of the DUF5396 domains in P75324 and P75327 with the OppA protein of *Bacillus subtilis* showed very high scores and good structural similarity (Fig. 2D). This suggests that DUF5396-containing proteins function in oligopeptide binding.

### IDR content correlates with protein degradation rates

**Fig 3:**
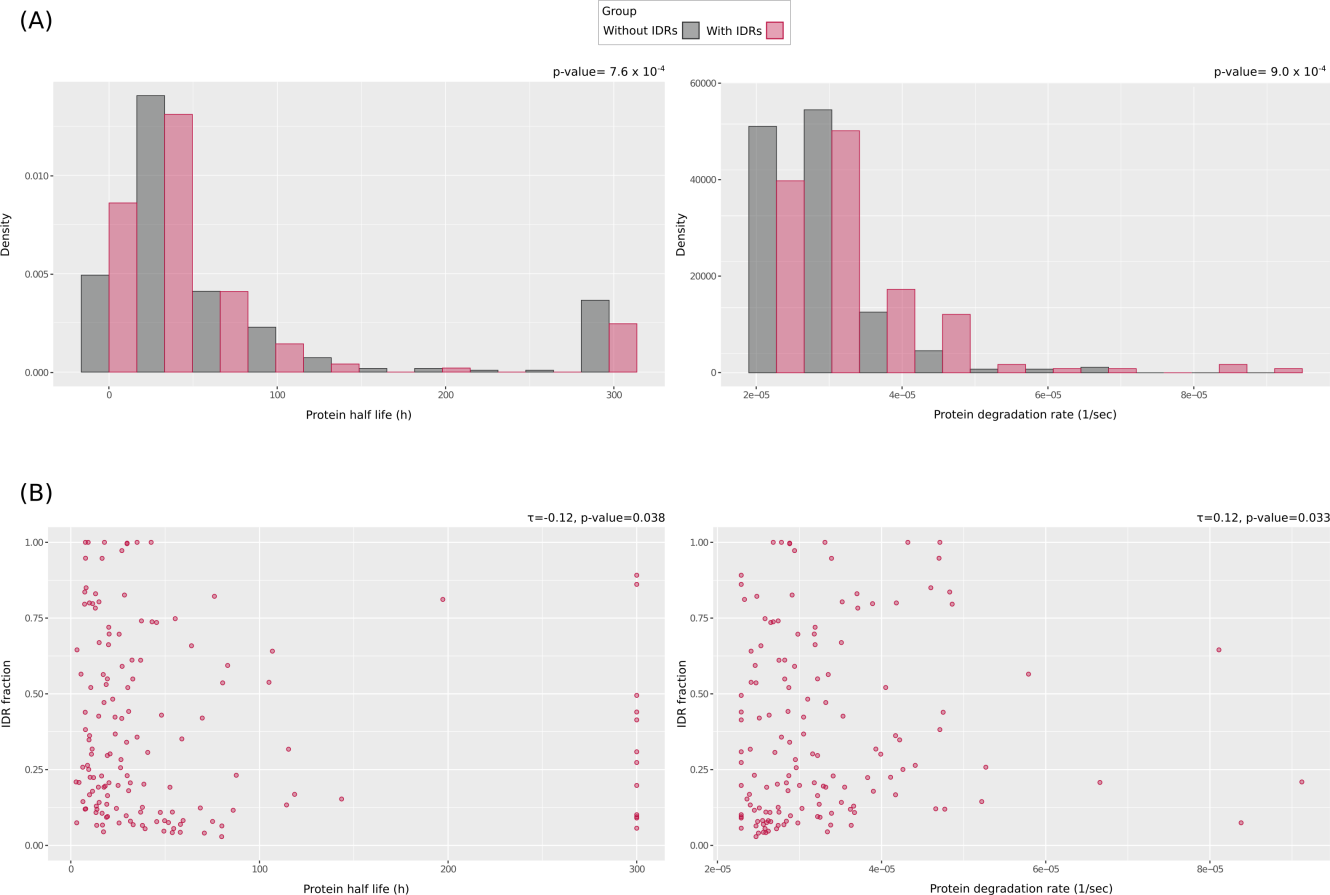
Analysis of protein half-life and degradation rates in relation to structural disorder. (**A**) Density histograms of protein half-life (left) and degradation rates (right) for Mpn proteins that have or do not have IDRs. *P*-values are from Brunner-Munzel tests. (**B**) Kendall’s correlation between IDR fraction and protein half-life (left) or degradation rate (right). Only IDR-containing proteins were analyzed.

In eukaryotes and *Escherichia coli*, disordered regions affect protein degradation rates (38–40). To assess whether this was also the case in Mpn, we exploited data from two recent works that measured protein abundance, half-life, degradation rate, and translational efficiency for ~470 proteins that were also included in our data set (41, 42) (Table S2). When proteins with IDRs were compared to those without IDRs, no differences were observed in protein abundance or translational efficiency (not shown). However, proteins with IDRs were found to have significantly shorter half-lives than proteins with no IDRs (Brunner-Munzel test, *P*-value = 7.6 × 10^−4^) (Fig. 3A). Because protein half-life and degradation rates are inversely related (Pearson correlation coefficient = 0.49, *P*-value < 2 × 10^−16^), IDR presence was also associated with significantly faster degradation (Brunner-Munzel test, *P*-value = 9.0 × 10^−4^) (Fig. 3A). To determine whether these effects were secondary to the functional or biochemical characteristics of IDR-containing proteins, we restricted the analysis to proteins with IDRs (Fig. 3B). A significant negative correlation between half-life and IDR fraction was observed, while the correlation with degradation rates was significant and positive (Fig. 3B). It should, however, be noted that the correlations are weak and should be treated with caution. In any case, these results suggest that structural disorder affects protein stability in Mpn by increasing degradation rates.

### IDRs are preferential sites of phosphorylation, but not of acetylation or proteolytic cleavage

**Fig 4:**
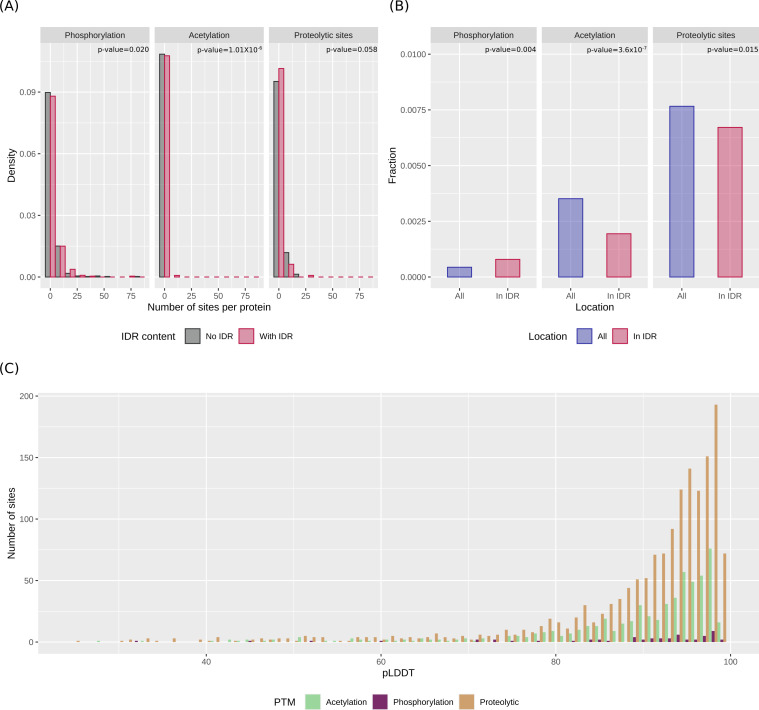
Analysis of PTMs in relation to structural features. (**A**) Density histograms of the number of phosphorylation, acetylation, and proteolytic sites in Mpn proteins that have or do not have IDRs. *P*-values are from Exact binomial tests. (**B**) Fraction of sites in IDRs compared to all PTMs. *P*-values are from Exact binomial tests. (**C**) pLDDT scores for all phosphorylation, acetylation, and proteolytic sites that are not located within IDRs.

In the human proteome, IDRs were shown to be common targets of post-translational modifications (PTMs) (43). It is unknown whether this is also the case for IDRs in prokaryotes. To explore this issue, we exploited data from a study that profiled phosphorylation and lysine acetylation in 460 Mpn proteins, 459 of which were present in our data set (44). We first asked whether, among these proteins, those with IDRs had a different number of PTMs than those with no IDRs. We found that IDR-containing proteins carry more phosphorylation sites (Exact binomial test, *P*-value = 0.020) and significantly fewer acetylated lysines (Exact binomial test, *P*-value = 1.01 × 10^−6^) (Fig. 4A) (Table S2). We next moved to the site level and investigated the localization of PTMs with respect to IDRs and structured domains. Results indicated that, out of 79 phosphorylation sites, 22 are located in IDRs, a number significantly higher than expected given the proportion of sequence embedded in IDRs (Exact binomial test, *P*-value = 0.004) (Fig. 4B). The opposite was true in the case of acetylated lysines, with fewer than expected being located in IDRs (54 in IDRs out of 634, Exact binomial test, *P*-value = 3.6 × 10^−7^) (Fig. 4B).

Mpn was shown to use a mechanism of controlled post-translational cleavage as a strategy to diversify its proteome (45). Recently, about half of Mpn proteins were found to undergo proteolytic processing, and the cleavage sites were identified (46). We thus hypothesized that IDR-containing proteins are preferentially cleaved, but we found no difference in the number of cleavage sites between IDR-containing and IDR-lacking proteins (average number of cleavage sites = 2.7 and 2.6, Exact binomial test, *P*-value = 0.58) (Fig. 4A) (Table S2). Surprisingly, at the site level, we found that proteolytic cleavage is less likely to occur in IDRs than in structured domains (281 in IDRs out of 1835, Exact binomial test, *P*-value = 0.015) (Fig. 4B).

In the human proteome, PTMs were shown to be particularly abundant in short IDRs (fewer than 20 AA in length) (43). We thus hypothesized that the same might happen in Mpn and that our cutoff for the definition of IDRs at 30 AA affected the conclusion that acetylation and proteolysis preferentially occur in structured domains. We thus examined the pLDDT scores of all sites involved in PTMs that are not located in IDRs. We found that the overwhelming majority of such sites have pLDDT scores higher than 80, indicating that they are not located in short IDRs or flexible loops (Fig. 4C).

### Conformational properties of Mpn IDRs

**Fig 5:**
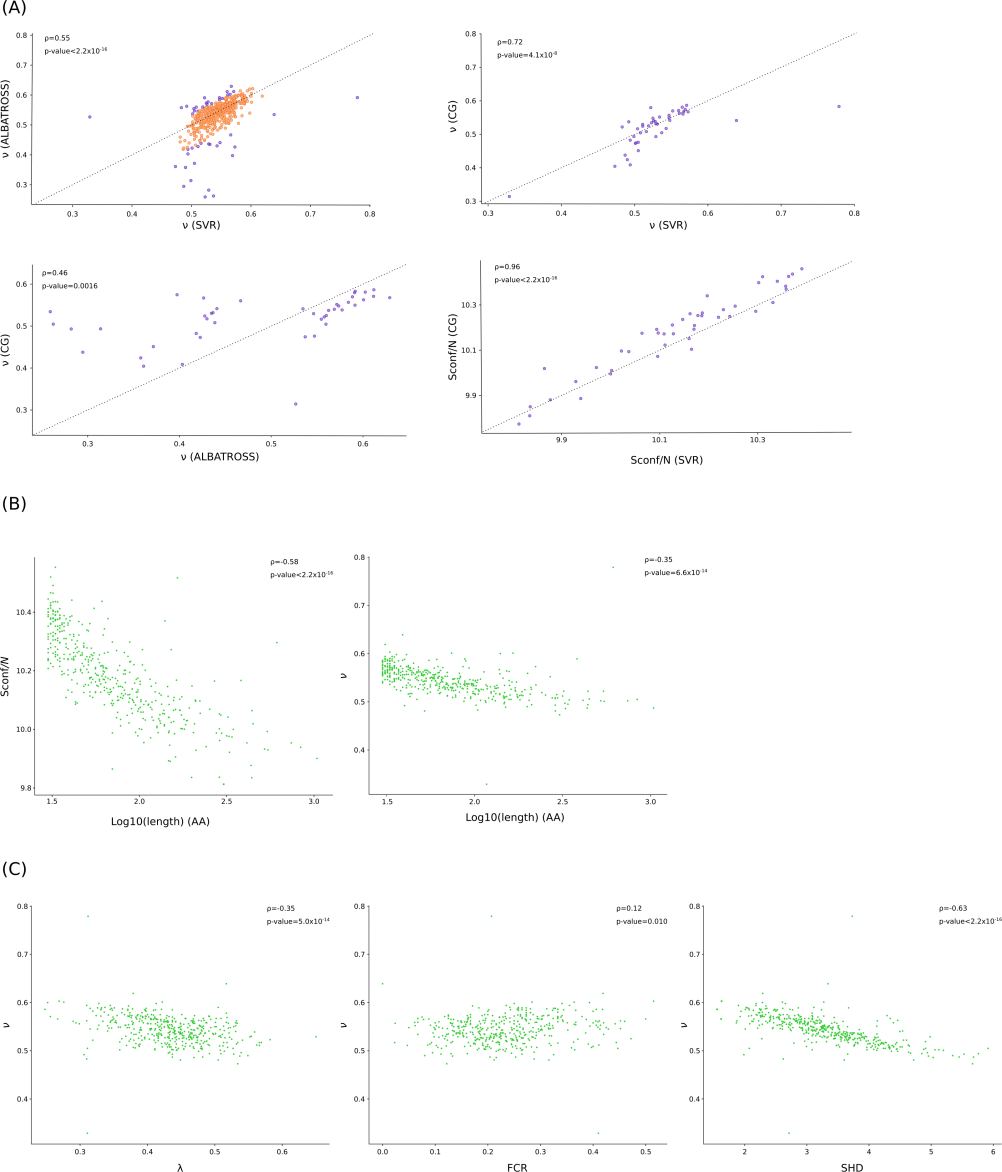
Conformational properties of Mpn IDRs. (**A**) Pearson’s correlation of estimates of the Flory scaling exponent (*ν*) obtained with ALBATROSS and with the SVR predictors. 44 IDRs that are at the extremes (5th and 95th percentiles) in the distribution of the difference between predictors are shown in purple. These IDRs were analyzed using coarse-grained (CG) simulations, and results are shown in comparison with those obtained from the SVR predictor or from ALBATROSS. Comparison between conformational entropy per residue (Sconf/N) estimates from CG simulations and those obtained from the SVR predictor is also shown. (**B**) Pearson’s correlation between Sconf/N or ν values (from the SVR predictor) with IDR length. (**C**) Pearson’s correlation between Sconf/N or ν values (from the SVR predictor) with different sequence features: average residue stickiness (λ), sequence hydropathy decoration (SHD), and fraction of charged residues (FCR).

We next aimed to investigate the biophysical properties of IDRs in the Mpn proteome. Although they do not adopt fixed structures, IDRs can be described through their ensemble features. These include the Flory scaling exponent (*ν*), a length-independent measure of chain compactness, as well as other parameters such as the conformational entropy per residue (*S*conf/*N*) (1). Recently, two predictors of IDR conformational properties were developed. One of them is based on a support vector regression (SVR) model and was trained on coarse-grained (CG) simulations of all the IDRs in the human proteome (3). The other, ALBATROSS, is a deep-learning model trained on GC simulations of naturally occurring IDRs mostly from eukaryotic proteomes, but also from prokaryotic ones, plus a library of synthetic sequences (47). Because none of the predictors was specifically trained on bacterial IDRs, we used both to estimate *ν* for all IDRs in the Mpn proteome (Table S4). Comparison of the estimates revealed a generally good correlation, although some IDRs deviated sensibly (Fig. 5A). We thus selected 44 IDRs that were at the extremes (5th and 95th percentiles) in the distribution of the difference between predictors to run CG simulations using the CALVADOS 2 force field (3, 48). Results showed that the estimates of ν from the SVR predictor were more similar to the ones from the CG simulations compared to those obtained with ALBATROSS (Fig. 5A; Table S4). A strong correlation was also detected for Sconf/N between the SVR predictor and the CG simulations (this parameter is not implemented in ALBATROSS) (Fig. 5A). It is worth mentioning that the SVR predictor was trained on simulations based on the CALVADOS 2 force field, possibly explaining its better fit with our CG simulation results. However, for IDRs in the human proteome, comparison of CALVADOS2 simulations with ALBATROSS predictions showed a very high correlation (*R*2 coefficient =  0.98) (47). Thus, these data suggest that the investigation of IDR conformational properties benefits from the comparison of different methods, especially in organisms whose IDRs are poorly studied and not included in predictor training. Also, it is worth noting that even the agreement between the SVR predictor and the CG simulations is good, but far from perfect, further supporting the need for more extended training data.

Given the results above, we performed all the following analyses using the estimates obtained with the SVR model. As is the case in the human proteome, we observed that long IDRs tend to be more compact and have lower conformational entropy (Fig. 5B) (3). To assess the sequence features associated with IDR compaction, we next used the SVR predictor to calculate the following parameters: average residue stickiness (λ, a measurement of the strength of attractive intra-chain interactions [48]), sequence hydropathy decoration (SHD, a measure of the patterning of hydrophobic residues [49]), and fraction of charged residues (FCR) (Table S4). Again, in line with data from the human proteome, we found that compact IDRs tend to have high values of λ and more clustered hydrophobic residues (high SHD), as well as fewer charged residues (Fig. 5C) (3). Overall, these results suggest that even if the representation of IDRs in the Mpn proteome is much lower than in human proteins, sequence-ensemble relationships tend to be conserved irrespective of whether proteins have a eukaryotic or prokaryotic origin. We finally sought to determine whether IDRs that are targets of PTMs are more or less compact compared to those that are not. To test this, we fitted linear regression models with *ν* as the dependent variable and IDR size and presence/absence of individual PTMs as independent variables. This approach was motivated by the fact that, although *ν* is a length-independent measure, we observed a relation with IDR size, and the probability of a PTM occurring in a given IDR will also depend on its length. Beyond the effect of length, we found a significant contribution of phosphorylation (estimate = 0.027, *P*-value = 0.0002), whereas the coefficients associated with acetylation and proteolysis were not significant (not shown). This indicates that phosphorylation preferentially occurs in extended IDRs.

### IDRs in attachment organelle proteins are extended and have high conformational entropy

The GO term “adhesion of symbiont to microvasculature” includes five contributing proteins (Fig. 1E), all localizing to the attachment organelle, which is, however, formed by at least 14 proteins, organized in different structures. We thus investigated whether the organelle proteins not assigned to the GO term related to adhesion also had IDRs (Table S2). We found this to be the case, as all of them, excluding CpsG, have at least one IDR. In fact, these additional nine proteins have a significantly higher IDR fraction than all other Mpn proteins (Brunner-Munzel Test, *P*-value = 0.002).

**Fig 6:**
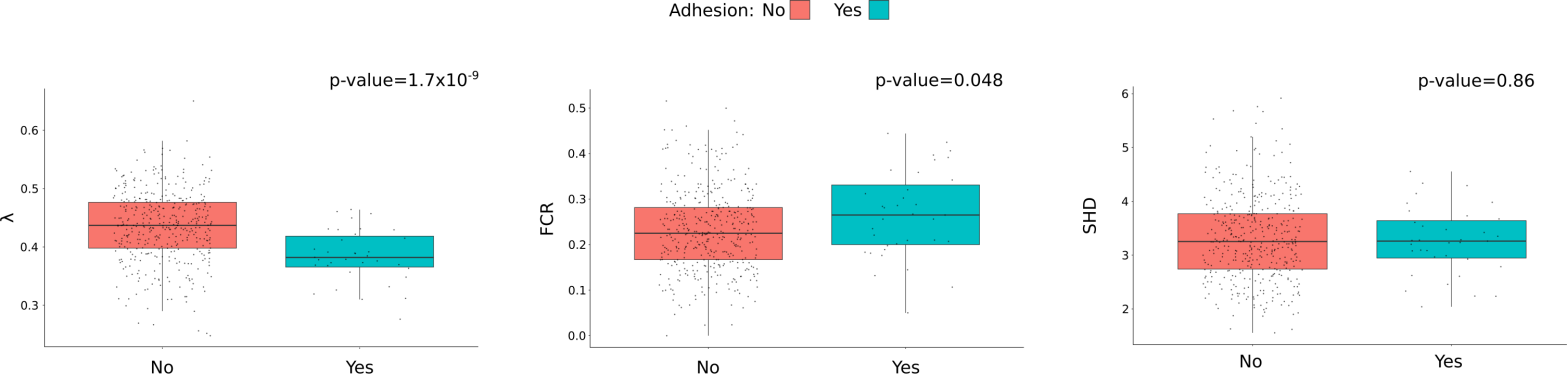
Analysis of IDRs located in attachment organelle proteins. Comparison of sequence features between IDRs that are located in attachment organelle proteins or in all other proteins. Data are shown in classic box and whisker plots. The *P*-values derive from Brunner-Munzel tests.

We next sought to determine whether IDRs in proteins that constitute the attachment organelle have distinctive conformational features. We again used linear regression models that account for IDR size and found that IDRs in attachment organelle proteins are more extended (estimate = 0.018, *P*-value = 0.0003) and have higher conformational entropy (estimate = 0.064, *P*-value = 0.0013) compared to those in other Mpn proteins. In line with the results above, compared to other IDRs in the Mpn proteome, IDRs in attachment organelle proteins have lower λ (Brunner-Munzel Test, *P*-value = 1.7×10^−9^) and higher FCR (Brunner-Munzel Test, *P*-value = 0.048). No difference in SHD was instead observed (Brunner-Munzel Test, *P*-value = 0.86) (Fig. 6).

## DISCUSSION

In this study, we performed a proteome-wide investigation of IDRs in Mpn, which leverages multiple levels of biological information. We found that, compared to other bacteria (25–27), a considerable fraction of the Mpn proteome is embedded in IDRs, which are particularly abundant in membrane, non-essential proteins, as well as in proteins that mediate cytoadherence and, hence, virulence. Notably, proteins that form the attachment organelle, a structure that is only found in Mpn and a few closely related mycoplasmas (*M. genitalium* and *M. gallisepticum*) (29), have an extremely high IDR content. Gliding motility in Mpn is not chemotactic, but contributes to the infection process, enabling the bacterium to translocate from the tips of bronchial cilia to the host cell surface (50, 51). Thus, these data suggest that structural disorder contributes to very specific functions in Mpn and, likely, in other mycoplasmas. In line with this view, we show that, in terms of domain architecture, IDR content is not particularly high in proteins that display nucleic-acid binding modules, such as transcription factors (as is instead the case in eukaryotes [13, 52]). Rather, IDRs tend to be preferentially associated with prokaryote- or Mollicute-specific domains. Among these, the DUF31 domain has been suggested to represent a determinant of pathogenicity (35), whereas the EAGR box, being located in attachment organelle proteins, is involved in gliding mobility (34). For another domain, DUF5396, despite its specificity for Mollicutes, we detected significant structural homology with the oligopeptide-binding proteins of other bacteria. These membrane-bound proteins are responsible for the uptake of peptides and, thus, for providing essential metabolites, with some also showing the ability to bind heme (53). It is thus possible that Mpn proteins carrying DUF5396 domains function in nutrient acquisition. Whether IDRs modulate essential features of these proteins, including binding specificity, or whether they contribute to nutrient sensing remains an open question for future investigation. Likewise, proteins carrying lipoprotein domains may act as transporters (33). However, lipoproteins in different *Mycoplasma* species have also been shown to regulate host cell immune response (54). Interestingly, some Mpn lipoproteins in family 2, which carry the lipoprotein X and lipoprotein 10 domains (33), and that we show here to have particularly high fractions of residues in IDRs, are differentially expressed when the bacterium gets in contact with human lung epithelial cells or in oxidative/acidic stress conditions (55). Thus, these proteins may play a role in adaptation to different environmental conditions and, possibly, contribute to virulence. Overall, these observations suggest that IDRs are common in Mpn proteins that either directly affect adherence, motility, and virulence or that play a role in the broader ability of the bacterium to survive and thrive within its host. We were also interested in determining whether, despite the fact that they are often located in proteins with mycoplasma-specific functions, IDRs in Mpn proteins share similar functional, biochemical, and conformational features with IDRs in other organisms. We found mixed evidence, depending on the analyzed features. For instance, we found that, as is the case for *E. coli*, yeast, and mammals, IDR content affects protein degradation rates and half-life (38–40). Protein degradation is mediated by different mechanisms in eukaryotes and prokaryotes. In the former, the covalent binding of ubiquitin marks proteins for degradation by the proteasome, whereas bacteria use ATP-dependent proteases for substrate degradation (56), and Mpn encodes only two such proteases (Lon and FtsH [41]). In eukaryotes, the lengths of the disordered regions that affect protein half-life were shown to be compatible with the structure of the proteasome (40). In bacteria, exposure of aromatic or hydrophobic residues in IDRs may promote degradation (41, 57). Thus, the shorter half-life of proteins with IDRs seems to be mediated by independent mechanisms in eukaryotes and prokaryotes*.* The two Mpn proteases belong to the AAA+ (ATPases associated with various cellular activities) family, hexameric enzymes that recognize specific substrates and use ATP hydrolysis to mechanically unfold the target protein and translocate it into the degradation chamber (58, 59). Initiation of degradation requires the recognition of a specific peptide signal (a degron) and the engagement of an unstructured segment of the substrate. The unstructured segment can be N-terminal, C-terminal, or internal (58–60). It is thus possible that the intrinsic lack of structure of IDRs facilitates engagement by the proteases and thus promotes protein degradation. In this respect, it is worth noting that we found a significant association between IDR presence and degradation rates, whereas the correlation between IDR fraction and protein degradation was weak. This suggests that the presence of unstructured segments rather than their length modulates protein turnover. An interesting possibility is that evolution has developed mechanisms to increase the degradation rate of IDR-containing proteins, possibly to control their aggregate-forming propensity. Just like IDRs, PTMs are less common in prokaryotes compared to eukaryotes. Our analysis of proteome-wide studies showed that, as in the case of eukaryotes, phosphorylation is common within Mpn IDRs, particularly those that assume an extended conformation, which may make them even more accessible to kinases (4,61,3, 61). This is interesting because phosphorylation, which equates to the gain of a negative charge, can impact IDR ensemble features and, as a consequence, properties such as binding affinity and protein interactions (1). Conversely, we did not find an increased frequency of acetylation or proteolytic processing in IDRs. Especially the latter finding was surprising, as IDRs are known to be intrinsically more susceptible to degradation, in line with the results we obtained for protein half-life (62). However, the controlled proteolysis of Mpn proteins, rather than serving purposes related to cellular homeostasis, is thought to function as a strategy to diversify the minimal proteome of the bacterium so that cleaved fragments can moonlight at the cell surface (45, 46). The products resulting from proteolysis were suggested to be more disordered than the original proteins, as it may be expected when cleavage disrupts the architectures of folded domains. Such products also engage in more protein-protein and protein-nucleic acid interactions, possibly due to the exposure of short linear motifs that are inaccessible within folded domains (46). Thus, if disorder is functional to the adaptive strategy of Mpn, it may be advantageous to use proteolytic sites within structured regions rather than in already disordered segments.

Recently, methods based on CG simulations and deep learning have allowed the large-scale analysis of IDR conformational properties, which are, in turn, related to function (1). To obtain ensemble features for Mpn IDRs, we used two methods, based on different force fields, which were shown to have very high concordance when human IDRs were analyzed (3, 47). Consistently, we found an overall good agreement between the two methods, although the correlation coefficient was not particularly high. We also observed that the prediction for some IDRs was quite divergent. Among these, the largest discrepancies were observed for IDRs that were predicted to be much more compact by ALBATROSS than by SVR. We thus used CG simulations to determine which predictions better fit the simulated ensembles. Specifically, we performed CG simulations under the CALVADOS model, and it is thus probably expected that we observed a better fit with the cognate predictor. However, some differences between the simulations and the predictor were still observable, and two IDRs that were predicted to have extremely extended ensembles (*ν* > 0.6) were instead found to be more compact with the simulations. Overall, these data indicate that, whereas the deep learning predictors provide an overall good picture of the ensemble properties of Mpn IDRs, some of the latter might fail to be accurately described using these approaches. More generally, our data call for a wider benchmarking of computational methods to predict IDR ensemble properties, especially when these are applied to prokaryotes or other organisms whose IDRs were not used for predictor training. These observations notwithstanding, our results consistently show that ensemble properties in Mpn IDRs are mediated by similar sequence features as in eukaryotes and that compact IDRs tend to have high residue stickiness, high hydropathy decoration, and few charged residues (3, 47).

Remarkably, when we analyzed IDRs in attachment organelle proteins, we found that they do not only cover a considerable portion of the protein sequence, but are also particularly extended and display high conformational entropy. The attachment organelle forms at a cell pole as a protrusion comprising surface nap-like structures (the P1/P90/P40 adhesin complexes) and an internal rod-like cytoskeletal core surrounded by a translucent area, which is thought to be occupied by stiff material. The nap-like structures interact with sialic acids at the target cell surface, and the source of energy is thought to derive from ATP hydrolysis, although the underlying mechanisms are unknown (29, 63, 64). Very recent evidence showed that, when the conformational entropy of an IDR is constrained, for instance by tethering to a surface or by confinement in periplasmic space, an effective entropic force is generated (65, 66). This mechanism is, for instance, exploited by Gram-positive bacteria to drive protein translocation through the cell wall in the absence of chaperones or energy sources (65). An interesting possibility is that a similar force is generated in the attachment organelle of Mpn by the IDRs, which we show here to have high conformational entropy. Their confinement by the stiff translucent area or other structures might generate an entropic force that contributes to movement. This hypothesis is supported by the observation that phosphorylation of proteins in the attachment organelle, which is, in turn, expected to modulate ensemble properties, affects gliding motility (67–69). It is also worth noting that IDRs were previously shown to contribute to flagellum-mediated swimming and swarming in *gammaproteobacteri*a (12). Compared to the attachment organelle, the flagellum has a completely different structure, and the small, disordered proteins that mediate motility in this system play unknown roles (12). However, structural disorder seems to have been exploited independently in distinct bacteria to promote different types of motility. Future studies will be necessary to investigate the role of IDRs and the overall prevalence of these sequences in proteins that mediate motility in bacteria.

In summary, our results indicate that structural disorder contributes to very specialized functions in Mpn and most likely affects virulence, motility, and nutrient scavenging. Our data also highlight the functional relevance of IDRs: despite genome reduction, whereby a number of pathways were lost or simplified, the minimal proteome of Mpn displays a considerable level of structural disorder.

## MATERIALS AND METHODS

### Proteome and disorder prediction

The reference proteome of Mpn strain ATCC 29,342/M129 (UP000000808) was downloaded from UniProt. Of 686 proteins, 685 were available in the AlphaFold structure database and had the same length as in UniProt (Table S2).

To identify IDRs, we used two methods: an AlphaFold2-based method, as recently reported in a study that analyzed the human proteome (3) and Metapredict V2 (23, 24). Briefly, we used the protti R package (70) to derive pLDDT scores from the AlphaFold structure database (https://alphafold.ebi.ac.uk/) (71). We next generated window-averaged pLDDT scores using a window size of 15 AA (72). Residues with ⟨pLDDT⟩  > 0.8 were considered as folded, and those with ⟨pLDDT⟩  < 0.7 were labeled as disordered. Residues with 0.7 ≤ ⟨pLDDT⟩  ≤ 0.8 were initially defined as gap regions. Next, folded and disordered regions shorter than 10 residues were reclassified as gaps. Gap regions were then reassigned to the disordered fraction if they were flanked by disordered regions on both edges or on one single edge when N- or C-terminal. All other gap regions were instead relabeled as folded. Disordered regions shorter than 30 residues were not considered IDRs (3) (Table S4).

The Metapredict tool defines IDRs by applying a deep-learning algorithm based on a consensus score calculated from eight different disorder predictors (23). Metapredict V2 was run using default parameters, and IDRs were defined as consecutive disordered stretches longer than 30 residues.

### Comparison with experimentally solved protein structures

To evaluate the performance of the AlphaFold-based method and of Metapredict, we compared the IDRs detected by the two approaches with experimentally solved structures, where regions that fail to be resolved (missing residues) are likely to represent IDRs (73). The structures of all available Mpn solved structures were downloaded from PDB (22) and are reported in Table S1. The PDB files were parsed to annotate the protein region that was used for structure definition and the missing residues (gaps). Gaps longer than 30 were considered IDRs. We next compared the gap locations from the PDB files with the AlphaFold and Metapredict IDR predictions. Specifically, we calculated specificity (TN/(TN + FP)), sensitivity (TP/(TP + FN)), and accuracy ((TP + TN)/(TP +TN + FP + FN)), where TP = true positive, TN = true negative, FP = false positive, and FN = false negative.

### Functional characterization of Mpn proteins

Data of protein essentiality were retrieved from a transposon insertion mutagenesis study (28). A list of virulence factors was derived from the MicroScope website using the VirulenceFinder tool with default parameters (https://mage.genoscope.cns.fr/) (30). Only experimentally validated hits were retained (Table S2). Information about Mpn protein complexes was obtained from a previous work (31). GO terms were derived from QuickGO (https://www.ebi.ac.uk/QuickGO/) using the protti R package (70) (Table S2).

### Protein domains, structure similarity search, and structural alignment

Domains were identified and annotated for all proteins in the Mpn proteome using the SMART batch access script (http://smart.embl-heidelberg.de/smart/batch.pl) with default parameters and inclusion of Pfam domains (74) (Table S3).

Structural models of P75324 and P75327 were obtained from the AlphaFold database (alphafold.ebi.ac.uk) (71). Foldseek (https://search.foldseek.com/) and DALI (http://ekhidna2.biocenter.helsinki.fi/dali/) were run using default parameters (36, 37, 75). For pairwise structure alignment, TM-align (76) was run from the dedicated website (https://zhanggroup.org/TM-align/) using the models of P75324 and P75327 and the resolved structure of the OppA protein from *Bacillus subtilis* (PDB ID: 8arn).

### Protein degradation rates, half-life, and PTMs

Measures of translational efficiency, protein abundance, degradation rates, and protein half-lives were derived from recent works (41, 42).

Data on phosphorylation and acetylation sites were retrieved from the study by van Noort and coworkers (44). Proteolytic processing data were derived from Reference (46).

### Analysis of IDR conformational properties and sequence patterns

The Flory scaling exponent (ν) was calculated for all Mpn IDRs identified using the AlphaFold2-based approach described above (77). ν was estimated using two methods: (i) a Colab notebook (https://colab.research.google.com/github/KULL-Centre/_2023_Tesei_IDRome/blob/main/IDR_SVR_predictor.ipynb), which uses a support vector regression model trained on simulations performed using the CALVADOS model (48, 78), and (ii) a second Colab notebook (https://colab.research.google.com/github/holehouse-lab/

ALBATROSS-colab/blob/main/example_notebooks/polymer_property_predictors.ipynb) that implements the ALBATROSS (A deep-Learning Based Approach for predicTing pRoperties Of diSordered proteinS) predictor (47). Both models predict conformational properties starting from IDR amino acid sequences. The SVR predictor was also used to derive other measures: conformational entropy per residue (*S*conf/*N*), the fraction of charged residues (FCR), the average residue stickiness (λ, a measurement of the strength of attractive intra-chain interactions), and the sequence hydropathy decoration (SHD, a measure of the patterning of hydrophobic residues) (Table S4).

### Molecular simulations

The conformational ensembles of IDRs were generated using the CALVADOS 2 coarse-grained model (3, 48). MD simulations were conducted under NVT conditions, employing the Langevin integrator with a 10 fs time step, and setting temperature and ionic strength at 310 K and 0.150 M, respectively. Both termini were modeled as charged, while histidine residues were kept in neutral form. MD simulations were performed for a total of 44 IDRs of lengths ranging from 32 to 840 residues, and depending on sequence length, the simulation time was calculated according to the guidelines of (3). More in detail, the total simulation steps are set as 1010*(number of saved frames), and converted to nanoseconds as ns = (number of steps)*0.01/1000. The number of saved frames is 7,000 for IDRs shorter than 150 residues, while for longer ones, it is calculated using the quadratic formula 3e^-4^*(number of residues)^2^*1,000. To improve sampling, four independent trajectories were simulated for each sequence. The properties of interest, ν and Sconf/N, were calculated as described in Reference (3), with their values averaged across the four replicas to ensure robust statistics.

### Statistical analysis

All statistical analyses were performed in the R v.4.0.5 environment. The Fisher’s exact test was used when comparing categorical data that can be structured as a contingency table (e.g., counts of proteins with or without IDRs in different essentiality classes). The test is robust to small samples. Pearson’s correlation was used to measure the degree of the relationship between linearly related variables. Kendall’s correlation is a non-parametric test that measures the strength of dependence between two variables based on the ranks of the data. Kendall’s correlation is reliable even in the presence of ties (79). The Exact binomial test compares the observed frequencies of the two categories of a dichotomous variable (e.g., being or not a PTM site) to the frequencies that are expected under a binomial distribution. The test is appropriate when the sample size is small compared to the population subject to inference. Linear models were used to analyze IDR features as a function of different parameters (e.g., belonging to the attachment organelle or not) accounting for size. The Brunner-Munzel test was performed using the brunnermunzel R package. This test is used to compare the distribution of a continuous variable (e.g., IDR fraction) between two groups. It was chosen because it is not influenced by tied values, and it does not require the assumption of equal variances between groups (80). Statistical details about each comparison can be found in the figure legends.

## Data Availability

Data supporting the findings of this study are available within the article and its supplemental material.

## SUPPLEMENTAL MATERIAL

Table S1 spectrum.00968-25-s0001.xlsx Mpn proteins with an experimentally solved structure and their not solved regions.

Table S2 spectrum.00968-25-s0002.xlsx List of proteins in the Mpn proteome.

Table S3 spectrum.00968-25-s0003.xlsx List of domains associated with IDRs.

Table S4 spectrum.00968-25-s0004.xlsx List of Mpn IDRs.

ASM does not own the copyrights to Supplemental Material that may be linked to, or accessed through, an article. The authors have granted ASM a non-exclusive, world-wide license to publish the Supplemental Material files. Please contact the corresponding author directly for reuse.
